# Breakage risk of different sacroiliac screws in unilateral sacral fractures a finite element analysis

**DOI:** 10.1186/s12891-022-05898-2

**Published:** 2022-11-04

**Authors:** Yupeng Ma, Yong Zhao, Dexin Zou, Shengjie Dong, Xiujiang Sun, Gong Cheng, Wei Lian, Yuchi Zhao, Tao Sun, Dan Wang, Shudong Zhang

**Affiliations:** 1 Orthopaedics Department, Yantai Shan Hospital, 91#, Jiefang Road, 264008 Yantai, Shandong Province P. R. China; 2 CT/MR Department, Yantai Shan Hospital, 91#,Jiefang Road, 264008 Yantai, Shandong Province P. R. China

**Keywords:** Pelvis, Sacral fracture, Sacroiliac screw, Screw breakage, Biomechanics

## Abstract

**Objective:**

To compare the breakage risk of lengthened sacroiliac screws and ordinary sacroiliac screws to treat unilateral vertical sacral fractures and provide a reference for clinical application.

**Methods:**

A finite element model of Tile C pelvic ring injury (unilateral type Denis II fracture of the sacrum) was produced. The sacral fractures were fixed with a lengthened sacroiliac screw and ordinary sacroiliac screw in 6 types of models. The maximal von Mises stresses and stress distributions of the two kinds of screws when standing on both feet were measured and compared.

**Results:**

The maximal von Mises stress of the lengthened screw was less than that of the ordinary screw. Compared with ordinary screw, the stress distribution in the lengthened screw was more homogeneous.

**Conclusions:**

The breakage risk of screws fixed in double segments is lower than that of screws fixed in single segments, the breakage risk of lengthened screws is lower than that of ordinary screws, and the breakage risk of screws fixed in S2 segments is lower than that of screws fixed in S1 segments.

## Introduction

Unilateral sacral fractures caused by high-energy injuries are rare [1], and most cases are accompanied by posterior ring injuries. Vertical fractures of the sacrum (AO type C3.1) are vertically unstable due to complete disruption of the posterior arch [2–4] and are accompanied by high morbidity and mortality [4, 5]. Many studies have shown that such fractures cause complications such as malunion, nonunion of bones, neurological dysfunction, low back pain, abnormal gait, and bowel/bladder problems, and surgical treatment can reduce long-term complications [6–9]. Therefore, surgical treatment is necessary at the early stage for cases with unstable sacral fractures. The operation’s goal is to reconstruct the stability of the posterior pelvic ring by reducing and fixing the sacral fracture.

Various methods for vertically unstable sacral injuries have been advocated, including transiliac rods [10], transiliac plates [11], percutaneous iliosacral screws [12, 13], and spinopelvic instrumentation [14, 15]. In posterior pelvic ring injury treatment, sacroiliac screw internal fixation technology is commonly used [16]. Sacroiliac screws have significant biomechanical advantages and minimally invasive percutaneous puncture advantages in treating longitudinal fractures of the sacrum [17].

To maximize the effect of sacroiliac screws, we performed research [18–20] on ordinary sacroiliac screws and lengthened sacroiliac screws using radiological anatomical and biomechanical methods. Our previous studies have shown that the fixation effect can be increased when using lengthened sacroiliac screws and fixing S1 and S2 [19, 20]. However, few studies have assessed the breakage risk of lengthened sacroiliac screws and the factors involved in screw breakage. Thus, it is unclear whether an increase in screw breakage risk accompanies an increase in screw length. In other words, what is the relationship between the length of sacroiliac screws and mechanical safety performance? Solving this problem is of great value in guiding the application of sacroiliac screws in unilateral sacral fractures. However, no related studies have been reported.

This study aims to make a biomechanical comparison of two kinds of sacroiliac screws in various modes for fixing unilateral longitudinal sacral fractures using the three-dimensional finite element technique, assess factors involved in screw breakage and provide a theoretical basis for clinical practice.

## Methods

**Fig. 1:**
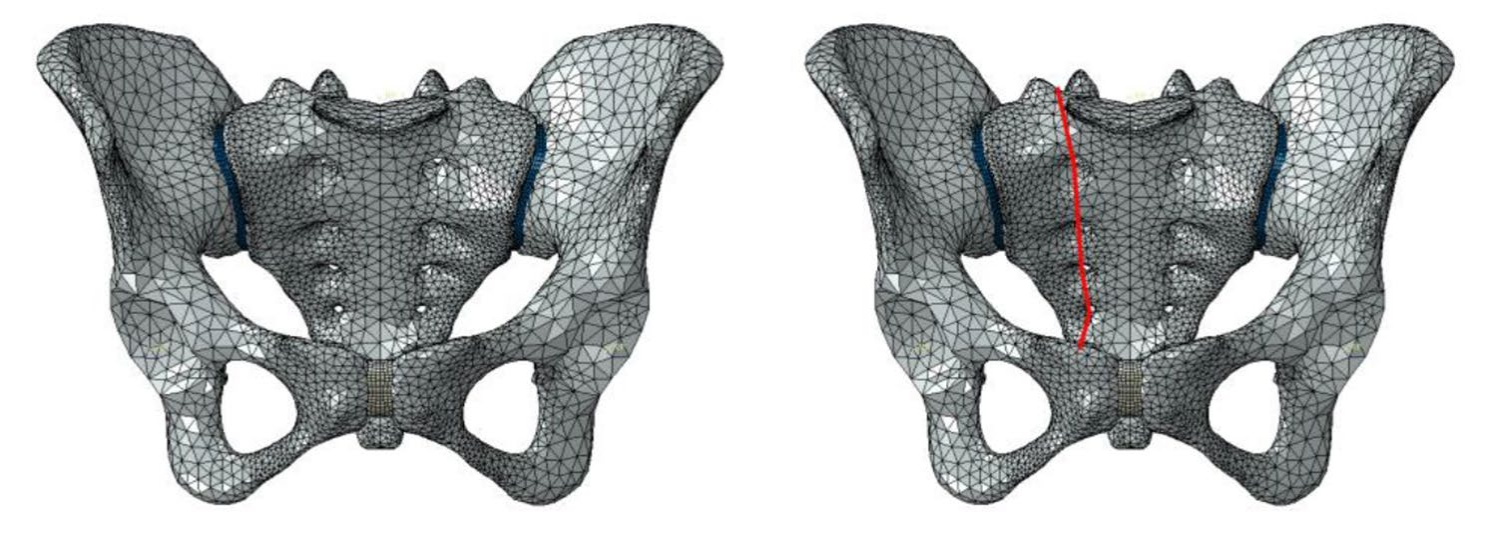
Pelvic three dimension finite element model. (The left is normal pelvic and the right is unilateral sacral fracture)

The finite element method [20] was adopted in this research, which we established in our earlier studies. (Fig. 1) A vertical 600 N load was loaded on the superior surface of the sacrum to simulate a standing human. ABAQUS 6.9.1 (SIMULIA, USA) software was used to extract mechanical simulation data from all models. We compare and measure the stress distribution and von Mises stresses of the two kinds of sacroiliac screws in various modes. The material parameters used in the model are shown in Tables 1 and 2 [21–23].

**Table 1:** Model parameters of pelvic ligaments

Ligament	K(N/m)	Number of springs
anterior and capsule	700	27
posterior (inner layer)	1400	15
intro-osseus	2800	8
sacrospinous	1400	9
sacrotuberous	1500	15
superior pubic	500	24
arcuate pubic	500	24

**Table 2:** Model parameters of various kinds of material

	Young’s modulus(MPa)	Poisson’s ratio	Element Type
cortical bone	18,000	0.3	shell element
trabecular bone	150	0.2	tetrahedral element
sacroiliac cartilage	1000	0.3	hexahedral element
interpubic disc	5	0.45	hexahedral element
screw	114,000	0.3	hexahedral element

Finite element models were constructed from computed tomography (64-slice spiral CT (Philips)) images of a normal female (36 years old, 170 cm, 63 kg). The slices were 1 mm thick. A virtual 3D model of the sacrum and innominate was created from CT data in DICOM format with image processing software (mimics 10.0). The geometric extents of pelvic cortical and trabecular bones were defined based on the surface mesh. By using four noded linear solid tetrahedral elements with an average edge length of 2 mm, we created an unstructured mesh of bone trabeculae in Abaqus/CAE. A triangular shell element represents the cortical bone surrounding the trabecular bone. The thickness of the shell element is 2 mm [21]. This setting is a homogeneous model setting and is more suitable for comparative studies.

We assumed a tied condition between the inner surface of cortical bone and the surface of the trabecular bone. The Young’s modulus and Poisson’s ratio were taken to be 150 N/mm^2^ and 0.2 for trabecular bone and 18,000 N/mm^2^ and 0.3 for cortical bone [18]. The sacroiliac cartilage and interpubic disc were continuum structures occupying the interspace and meshes into hexahedron elements. Binding was used between the sacrum, iliac bone, and sacroiliac joint, and bilateral pubis and pubic symphysis: sacroiliac ligament, sacrospinous ligament, and attachment regions were constructed according to a previous study [20]. The bilateral hip joint surface was coupled with a rotating centre. The centre point was constrained by translational degrees of freedom in three directions, simulating the mechanical properties in a standing state.

We cut the sacrum halfway through the right sacral foramina to construct a unilateral sacral fracture (AO type C3.1, Denis II) model. The final finite element normal pelvis and unilateral sacral fracture are shown in Fig. 1. In the simulation, we used two 7.3 mm cannulated screws (lengthened sacroiliac screw and sacroiliac screw) placed in either the S1 segment or S2 segment or both the S1 and S2 segments in a unilateral sacral fracture model. The material for the set screw was titanium alloy. The lengthened sacroiliac screw refers to the length of the sacroiliac screw that can pass through the contralateral sacroiliac joint and the contralateral iliac bone. Ordinary sacroiliac screws were set in this study to reach the midline of the sacrum. The proximal and distal ends of the screw were bonded to the bone tissue to simulate complete osseointegration, and the frictional relationship between the smooth rod part of the screw and the bone tissue was set as sliding.

**Fig. 2:**
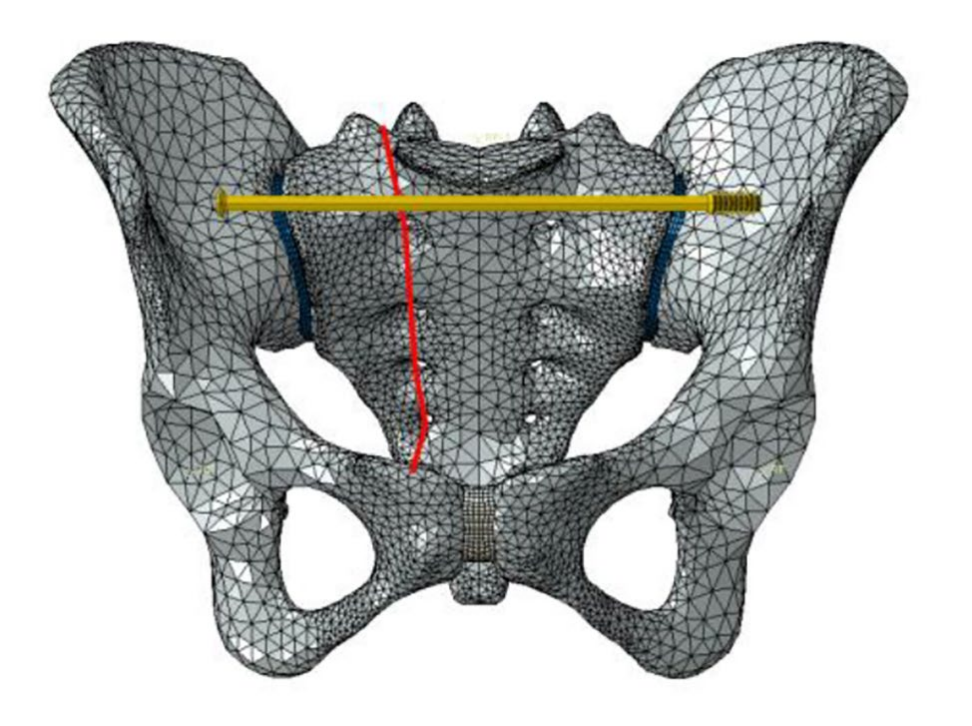
Sketch map of LS1

**Fig. 3:**
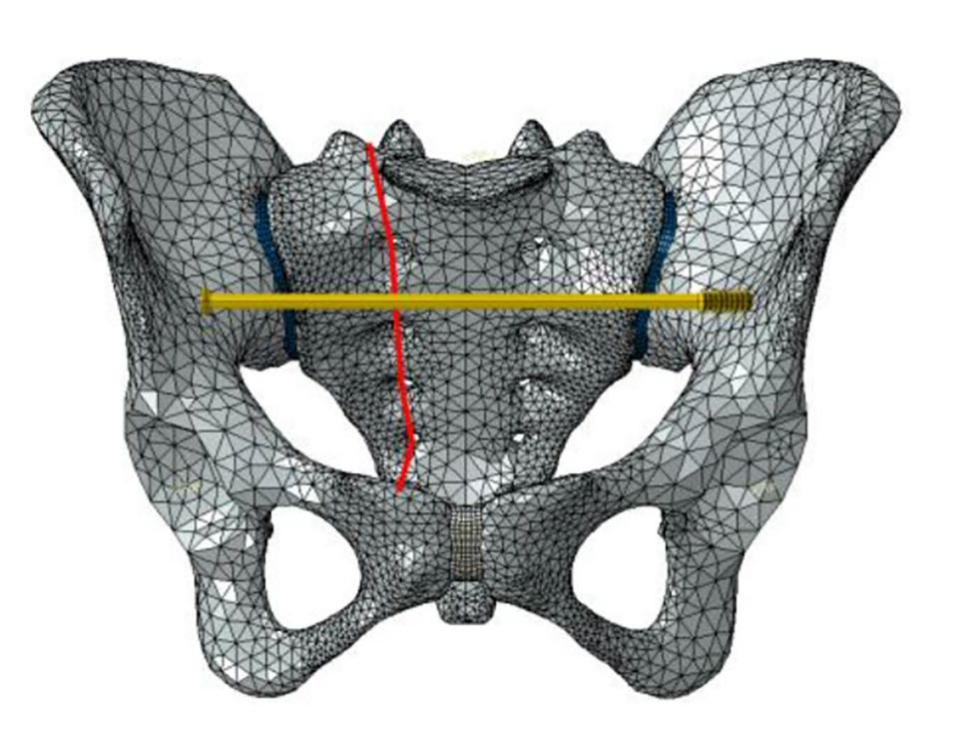
Sketch map of LS2

**Fig. 4:**
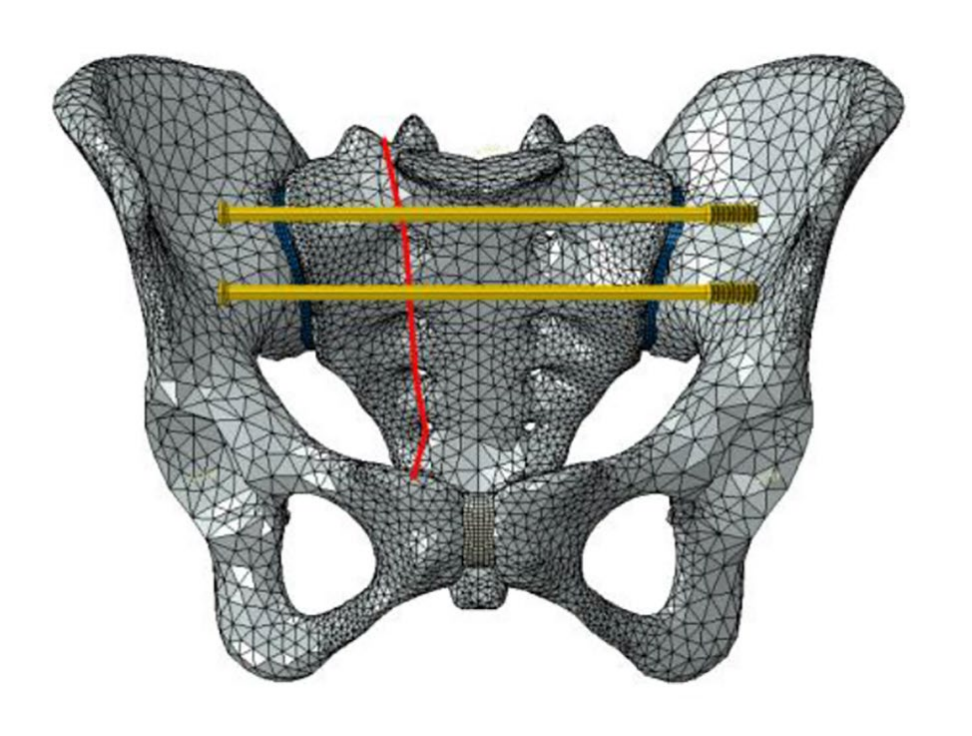
Sketch map of LS12

**Fig. 5:**
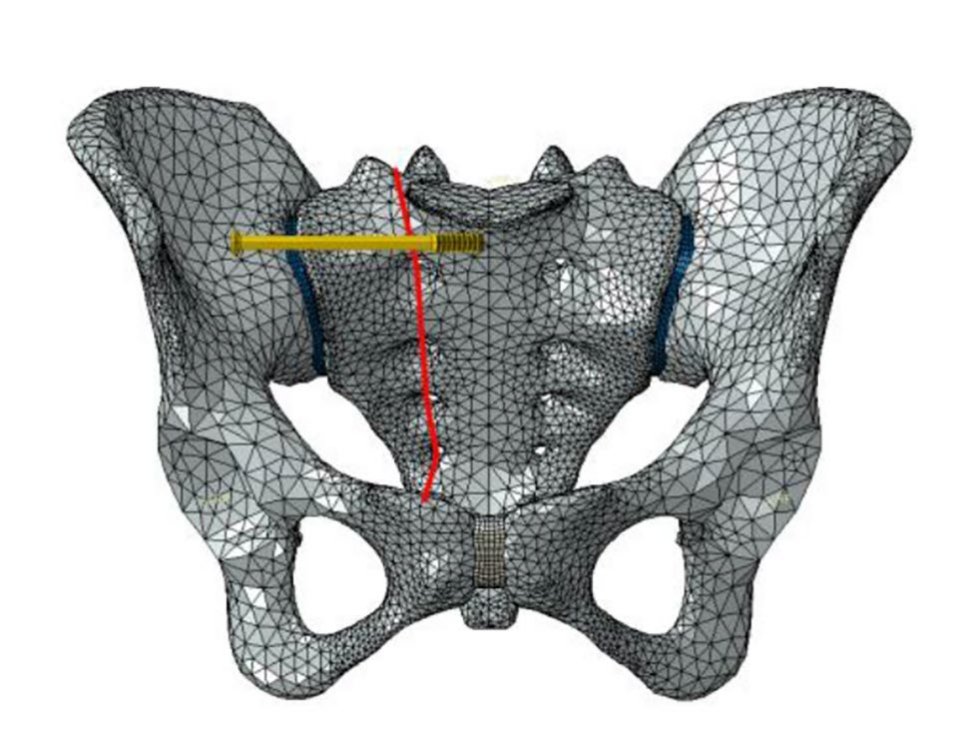
Sketch map of OS1

**Fig. 6:**
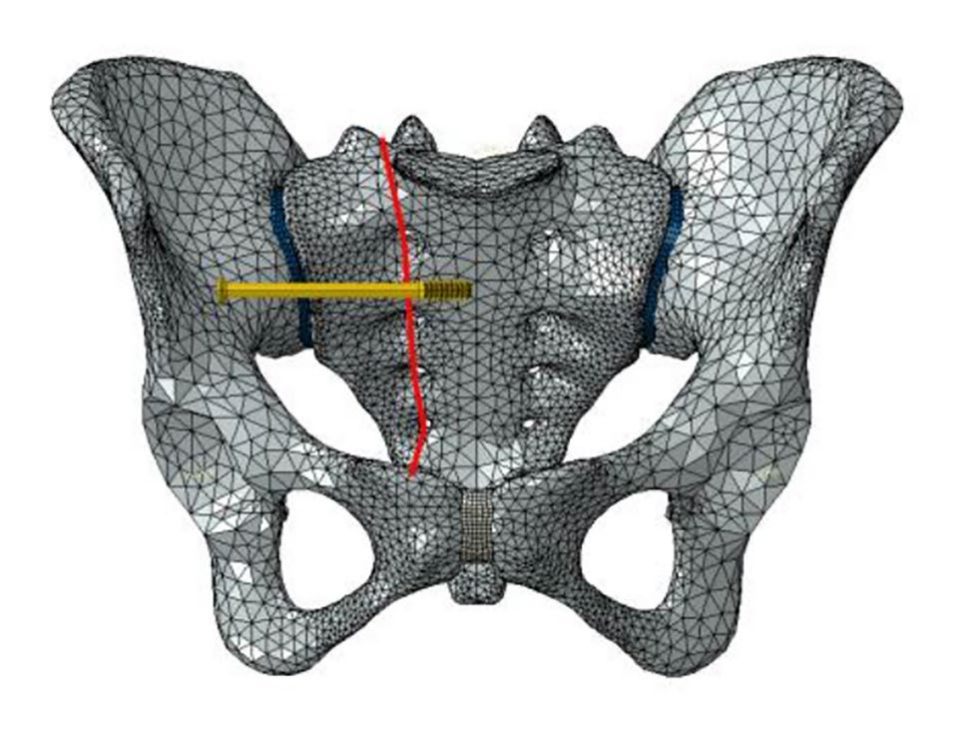
Sketch map of OS2

**Fig. 7:**
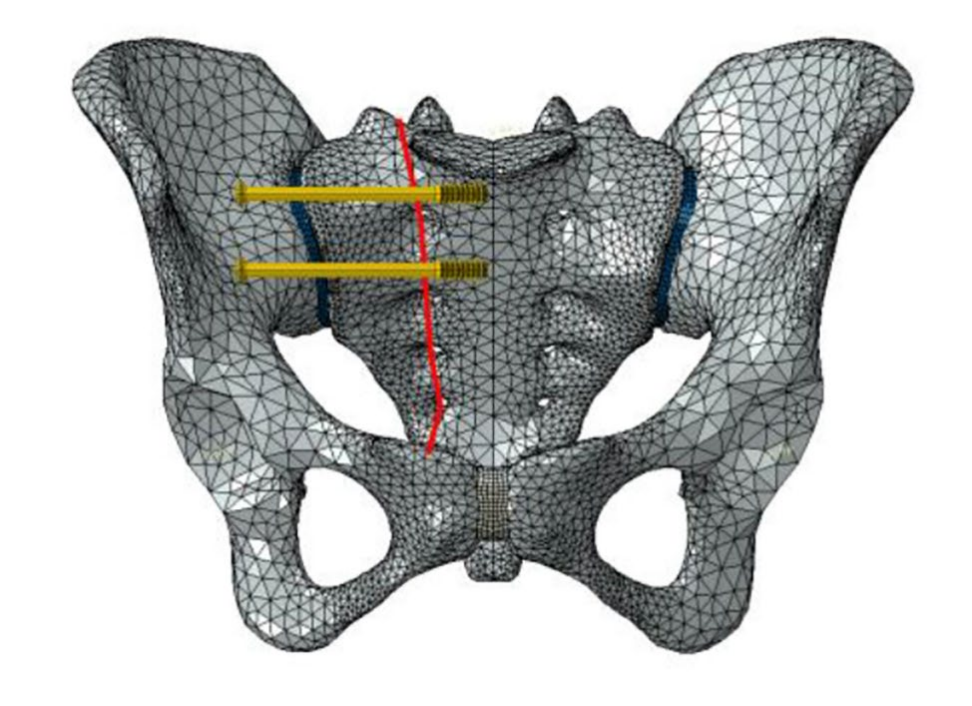
Sketch map of OS12

Six fixation cases were imitated for finite element analysis: ① one lengthened sacroiliac screw fixation in S1 segment (LS1) ② one lengthened sacroiliac screw fixation in S2 segment (LS2) ③ one lengthened sacroiliac screw fixation in S1 and S2 segments respectively(LS12) ④ one ordinary sacroiliac fixation in S1 segment (OS1) ⑤one ordinary sacroiliac fixation in S2 segment (OS2) ⑥ one ordinary sacroiliac fixation in S1 and S2 segments respectively (OS12) (Fig. 2–7).

Assembly was accomplished by placing constraints between interacting surfaces. These interaction surfaces were located at the bone–implant interface between the sacrum, the sacroiliac cartilage and the ilium, the pubic rami and interpubic disc, and the bone–implant interfaces in the screw thread. In the screw stem regions, frictionless sliding contact was used between the interaction surfaces of the bone–implant interfaces. We apply a penalty contact with a coefficient of friction of 0.3 on the fracture interaction surface. They were used to simulate sliding patterns between fracture interaction surfaces. The acetabular rotation centre was taken as the fixed point to fix the acetabulum to simulate the mechanical transfer of bipedal standing.

## Results

The results of this study show the following:

- In either model, the stress concentration area of the screw was found in the corresponding screw area of the sacral fracture area.
- The stress distribution of the lengthened sacroiliac screw was more homogeneous than that of the ordinary sacroiliac screw.
- The screw stress distribution in the model of different fixed segments of the same screw was more concentrated in the fixed single sacral segment than in the fixed two sacral segments.
- When both sacral segments were fixed, the distribution of screw stress in the upper segment was more concentrated, and the distribution of screw stress in the next stage was more uniform.
- The maximal von Mises stress of one sacroiliac screw fixation in the S1 or S2 segment was greater than that of one lengthened sacroiliac screw fixation in the same sacral segment;
- The maximal von Mises stress of one sacroiliac screw fixation in the S1 and S2 segments was greater than that of one lengthened sacroiliac screw fixation in the S1 and S2 segments;
- The maximal von Mises stress of one lengthened sacroiliac screw fixation in the S1 or S2 segments was greater than that of one lengthened sacroiliac screw fixation in the S1 and S2 segments;
- The maximal von Mises stress of one sacroiliac screw fixation in S1 or S2 segments was greater than that of one sacroiliac screw fixation in S1 and S2 segments (Table 3).

**Table 3:** The maximal Von Mises stress of screws (MPa)

	S1 segment	S2 segment
LS1	55.61	
LS2		49.61
LS12	36.98	38.85
	**S1 segment**	**S2 segment**
OS1	70.49	
OS2		50.77
OS12	46.49	44.14

## Discussion

Sacral fracture is a common occurrence in pelvic ring injury. The most common type is a unilateral sacral injury with anterior impaction of the sacrum, a lateral compression type 1 (LC-1) injury, which is usually associated with posterior ring instability and requires clinical treatment. A sacroiliac screw is a conventional internal fixation technique for posterior pelvic ring injury [16]. Sacroiliac screws are commonly used to stabilize the posterior ring. However, some clinical studies have suggested that conventional sacroiliac screw fixation may not provide sufficient stability universally. Keating et al. [24] applied sacroiliac screws to achieve 84% anatomic or near-anatomic reduction of pelvic fractures, but the final malunion rate was 44%. Damian et al. [12] showed that sacroiliac screws are clinically unreliable for vertical sacrum fractures. More recently, lengthened sacroiliac screws have come into use.Our paper described these as “lengthened sacroiliac screws.“ The screw is inserted from the external surface of the ilium across the contralateral sacroiliac joint and exits the ilium. Gardner et al. [25] described the advantages and theoretical basis of using a lengthened sacroiliac screw. First, lengthening sacroiliac screws has the characteristics of better vertical shear load distribution, lower tip stress, and resistance to displacement. Second, in addition to the absolute length of the screw, the lengthened sacroiliac screw allows for more threads to bind to the bone, which may increase holding power. Third, the lengthened sacroiliac screw provides anchorage in the iliac cortical bone, which may increase the role of the screw in maintaining reduction. Our sacral fractures based on biomechanical investigations [19, 20] showed that lengthened sacroiliac screws provide better stability than ordinary screws. However, it was not previously reported whether an increased risk of breakage accompanies the application of lengthened sacroiliac screws.

The maximum von Mises stress, one of the fundamental safety indicators of screws, increased with increasing screw fracture risk. The higher the stress is, the greater the likelihood of screw failure. The following results can be summarized from this study. When comparing different fixation modes with the same kind of screws, we found that the maximum von Mises stress was the largest, with only fixed S1 segments, and the minor model, with only fixed S2 segments. At least in the model, S1 and S2 are both fixed. Second, when fixing the S1 segment and S2 segment simultaneously, the maximum von Mises stress of the S1 segment screw in the same model was similar to that of the S2 segment screw, regardless of whether it was a lengthened screw model or an ordinary screw model. Third, if considered from the fixed segment, the screw fracture risk of double-segment fixation was lower than that of single-segment fixation. In double-segment fixation, the fracture risk of the two screws was similar. When different screws were used to compare the same fixed segment, we found that the maximal von Mises stress of the lengthened screw was lower than that of the ordinary screw, and the lengthened screw had a lower fracture risk than the ordinary screw.

In summary, from the perspective of screw safety, it is recommended to use lengthened screws for fixation. The safest method is to fix the S1 and S2 segments with extended sacroiliac screws. Normal sacroiliac screws are recommended for S1 and S2, even in the absence of lengthening sacroiliac screws. If only one screw can be used, S2 segment fixation is recommended regardless of the sacroiliac screw. The results of the screw safety analysis are consistent with those of the stability analysis.

The risk of a fatigue fracture in internal fixation can be reduced by avoiding excessive concentration in certain parts through the uniform distribution of stress in internal fixation. However, in this study, when we compared the stress distribution, we found that the stress distribution of screws was not uniform in any model. Compared with the different kinds of screws we used, the stress distribution of the lengthened sacroiliac screw was more uniform. Compared with different fixed segments, the stress distribution of double-segment fixed screws was more uniform. Similarly, these findings are consistent with the stability analysis results and the maximum von Mises stress analysis.

The following points need to be noted in this study. First, although anterior ring instability is characteristic of type C pelvic ring injuries, considering the multiple fixation methods of anterior ring fractures may affect the stability of the posterior pelvic ring. This study did not imitate the anterior pelvic ring’s injury and fixation but only maintained the anterior pelvic ring’s normal state. The anterior pelvic ring had a slight effect on the stability of the posterior pelvic ring and did not affect our comparison of several study models. Second, to best simulate pelvic stability, we reserved multiple important pelvic ligaments in our research. Meanwhile, to eliminate any unpredictable forces that might affect the measurements, we did not simulate muscles to simulate the extra stability they would cause. Muscle parameters, joint data parameters, and joint flexibility settings will qualitatively influence the results. However, the calculation is too complicated to complete the experiment, so they are simplified [26, 27]. Third, it was not feasible to simulate all the features of comminuted sacral fractures accurately. In our study, we used a well-accepted method to imitate a unilateral sacral sagittal fracture through the unilateral sacral foramen, which is considered the typical type of simulated sacral fracture (Denis II). Moreover, our model method had a straight and smooth fracture, which facilitated the standardization of the model and did not affect the accuracy of mesh generation and subsequent calculation. Fourth, to best mimic the normal state of the pelvis while standing, we positioned the pelvis so that the upper surface of the symphysis pubis was aligned with the lower surface of the sacrum. Fifth, the finite element model we studied was bone independent, and our conclusions were theoretically applied to patients who still have cartilage.

## Conclusion

The breakage risk of screws fixed in double segments is lower than that of screws fixed in single segments, the breakage risk of lengthened screws is lower than that of ordinary screws, and the breakage risk of screws fixed in S2 segments is lower than that of screws fixed in S1 segments.

## Data Availability

The datasets generated or analysed during the current study are not publicly available due the finite element model belongs to the subject but are available from the corresponding author on reasonable request. **Ethics declarations**.
